# A Novel Damping Control of Grid-Connected Converter Based on Optimal Split-Inductor Concept

**DOI:** 10.3390/mi13091507

**Published:** 2022-09-11

**Authors:** Chao Chen, Liwu Gong, Wei Zhang, Tao Wu, Yixing Gu, Changli Shi

**Affiliations:** 1 Grid Pinghu Power Supply Company, Pinghu 314200, China; 2 Pinghu General Electric Installation Co., Ltd., Pinghu 314200, China; 3 Institute of Engineering, Chinese Academy of Sciences, Beijing 100190, China

**Keywords:** current source converter, LC filter, passive damping control, split inductor

## Abstract

In this paper, a grid-connected converter is investigated. Since the AC side of the grid-connected converter is the LC filter, there is a second-order system resonance problem, and the conventional passive damping control has an inherent limitation of excessive power loss. Based on the mathematical model, a new damping control method is proposed in this paper. It is compared with the traditional solution in terms of damping effect, power loss and system stability. The optimal inductor split ratio is also discussed. The theoretical analysis demonstrates that the proposed method can not only achieve almost the same damping effect as the conventional solution, but also reduce the power loss of the damping resistor. The experimental tests are carried out and the experimental results verify the effectiveness of the proposed method.

## 1. Introduction

**Figure 1:**
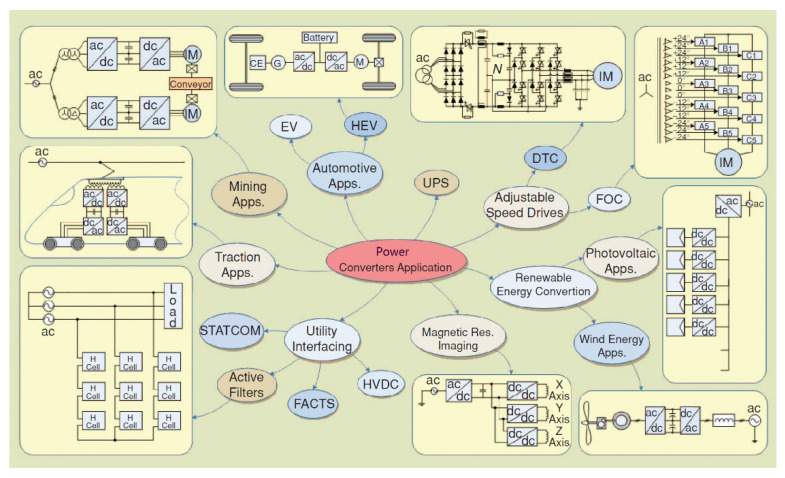
Power converters application.

The power converters have been widely used in the modern power system, as shown in Figure 1. Typically, the voltage source converter is used [1,2]. Another solution is to use the current source converter [3]. Since its AC side adopts the LC filter to achieve the high-frequency harmonics of the AC current, the LC structure will cause the system resonance phenomenon. It is necessary to take measures to increase the damping to suppress the potential resonance and enhance stability [4,5]. The active and the passive damping control scheme are two solutions to increase the system damping for stability improvement [6,7]. An active damping scheme based on the idea of “virtual resistance” introduces capacitive voltage or current into the closed-loop control [8], thereby suppressing the resonance peak of the system, and ensuring the system stability. In [9], the estimation method is used to introduce the high-frequency component of the inductor voltage into the control, while a complex high-pass filter is needed. In [10], the capacitive current feedback is used to suppress system resonance, and additional current sensors are needed. In [11,12], the filter capacitor voltage feedback method with series lead lag is proposed. However, the lead-lag module parameters need to be adjusted. In [13], an active damping method is proposed based on the genetic algorithm, which is complicated to implement.

On the other hand, the passive damping control achieves the resonance suppression by adding the resistance to inductors and capacitors, so as to enhance the system stability [14,15]. In [16], the four passive damping schemes are evaluated from the viewpoint of damping effect and power losses. The advantages and disadvantages of each passive damping scheme are discussed. Since the passive damping scheme has high damping power losses, the passive damping scheme of the inductive shunt resistor is designed for the current source converter in [17]. The active damping scheme does not introduce an additional power loss, but requires additional current or voltage sensors, increasing the complexity of the control algorithm and placing higher demands on the controller. The passive damping scheme is simple to implement and does not require additional sensors and control algorithms. However, it has the disadvantage of power loss, which will reduce the efficiency of the system.

In order to solve the abovementioned-problem, a novel damping control is proposed based on the optimal split-inductor concept. The major contribution of this paper is that the proposed method can not only achieve almost the same damping effect as the conventional one, but also reduce the power loss of the damping resistor. The experimental results are also provided and discussed. 

## 2. Mathematical Model Analysis

**Figure 2:**
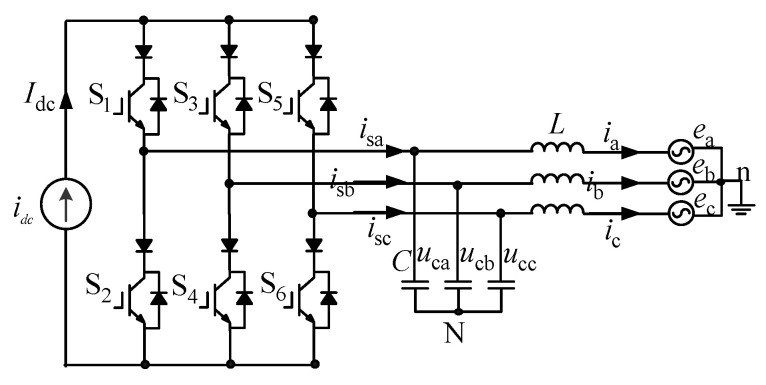
Schematic diagram of current source converter.

The schematic diagram of the current source converter is shown in Figure 2, where *i*_dc_ is a DC current source, *e*_a_, *e*_b_ and *e*_c_ are the instantaneous values of the three-phase grid voltage, *i*_a_, *i*_b_ and *i*_c_ are the instantaneous values of the three-phase grid current, *u*_ca_, *u*_cb_ and *u*_cc_ are the instantaneous values of the AC-side phase voltage, and *i*_sa_, *i*_sb_ and *i*_sc_ are the instantaneous values of the three-phase current. Inductor L and capacitor C form an LC filter to attenuate current high frequency harmonics. S1~S6 are (Insulated Gate Bipolar Transistor) IGBTs, where the additional diodes in series are used to provide reverse blocking capability [18,19,20].

The state equation is obtained using KCL and KVL laws, as shown in (1) and (2).

(1) $$C\frac{d}{dt}\left(\begin{array}{l} u_{\mathrm{ca}}\\ u_{\mathrm{cb}}\\ u_{\mathrm{cc}} \end{array}\right)=\left(\begin{array}{l} i_{\mathrm{sa}}\\ i_{\mathrm{sb}}\\ i_{\mathrm{sc}} \end{array}\right)-\left(\begin{array}{l} i_{a}\\ i_{b}\\ i_{c} \end{array}\right)$$

(2) $$L\frac{d}{dt}\left(\begin{array}{l} i_{a}\\ i_{b}\\ i_{c} \end{array}\right)+\left(\begin{array}{l} e_{a}\\ e_{b}\\ e_{c} \end{array}\right)+u_{\mathrm{nN}}\times I^{3\times 1}=\left(\begin{array}{l} u_{\mathrm{ca}}\\ u_{\mathrm{cb}}\\ u_{\mathrm{cc}} \end{array}\right)$$

where *u*_k_ and *i*_k_ are the instantaneous value of the AC-side capacitor voltage and the instantaneous value of the current, *i*_sk_ is the instantaneous value of the output bridge arm current of the converter, *u*_nN_ is the voltage between the midpoint of the grid and the midpoint of the capacitor, and $I^{3\times 1}$ is the unit matrix.

The following equation can be obtained by the KCL and KVL laws.

(3) $$i_{a}+i_{b}+i_{c}=0$$

(4) $$e_{a}+e_{b}+e_{c}=0$$

For the N point by KCL law, Equation (5) can be obtained.

(5) $$C\frac{du_{\mathrm{ca}}}{dt}+C\frac{du_{\mathrm{cb}}}{dt}+C\frac{du_{\mathrm{cc}}}{dt}=0$$

The three equations of Equation (2) are added to obtain that the midpoint n of the grid is equal to the point N of the intermediate point of the alternating current capacitor in the three-phase symmetrical system. Let the current source converter gain *K* be equal to the DC current.

(6) $$K=i_{\mathrm{dc}}$$

In summary, the system mathematical model in the three-phase abc coordinate system can be obtained, as shown in (7).

(7) $$\left\{ \begin{array}{l} C\frac{du_{\mathrm{ck}}}{dt}=i_{\mathrm{sk}}-i_{k}\\ L\frac{di_{k}}{dt}=u_{\mathrm{ck}}-e_{k}\\ K=i_{\mathrm{dc}} \end{array}\right.$$

According to the mathematical model, the switching transient process of the three-phase current source converter can be accurately described. Since the variables on the AC side of the model are all AC quantities in this coordinate system, the system analysis and the design of the controller are complex, and the mathematical model needs to be appropriately simplified. If the low-frequency model of the three-phase current source converter is established in the two-phase rotating dq coordinate system, the fundamental component of the AC side of the three-phase current source converter will be transformed into a DC component, so the analysis and design process of the system can be simplified.

The 3/2 transformation is used to transform the mathematical model from a three-phase stationary coordinate system to a two-phase dq coordinate system, which simplifies the analysis and design of the control system. Converting the AC variables in the stationary coordinate system to the DC variables in the dq coordinate system can reduce the number of system variables. In summary, the mathematical model of the system in the dq coordinate system is shown in (8).

(8) $$\left\{ \begin{array}{l} C\frac{du_{\mathrm{cd}}}{dt}=i_{\mathrm{sd}}-i_{d}+\omega Cu_{\mathrm{cq}}\\ C\frac{du_{\mathrm{cq}}}{dt}=i_{\mathrm{sq}}-i_{q}-\omega Cu_{\mathrm{cd}}\\ L\frac{di_{d}}{dt}=u_{\mathrm{cd}}-e_{d}+\omega Li_{q}\\ L\frac{di_{q}}{dt}=u_{\mathrm{cq}}-e_{q}-\omega Li_{d}\\ K=i_{\mathrm{dc}} \end{array}\right.$$

where all the values in this set of equations are refer to the d_q_-coordinate system.

## 3. Proposed Solution

### 3.1. Split Inductor Passive Damping Control

**Figure 3:**
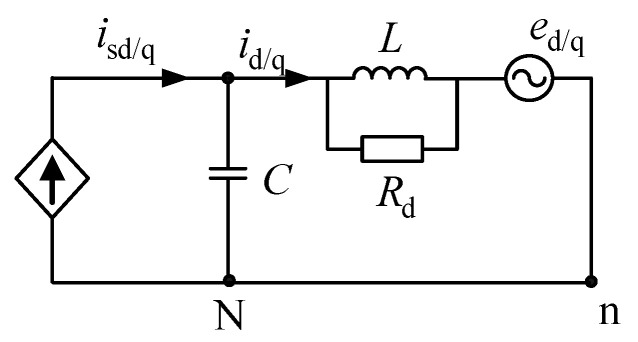
Traditional passive damping.

Traditional passive damping control adopts the damping scheme by paralleling a resistance to the inductor, as shown in Figure 3.

Ignoring the influence of the low-frequency grid, according to Figure 3, the transfer function of the passive damping control scheme of the conventional solution can be obtained as follows.

(9) $$\frac{i_{d/q}(s)}{i_{\mathrm{sd}/q}(s)}=\frac{Ls+R_{d}}{R_{d}CLs^{2}+Ls+R_{d}}$$

**Figure 4:**
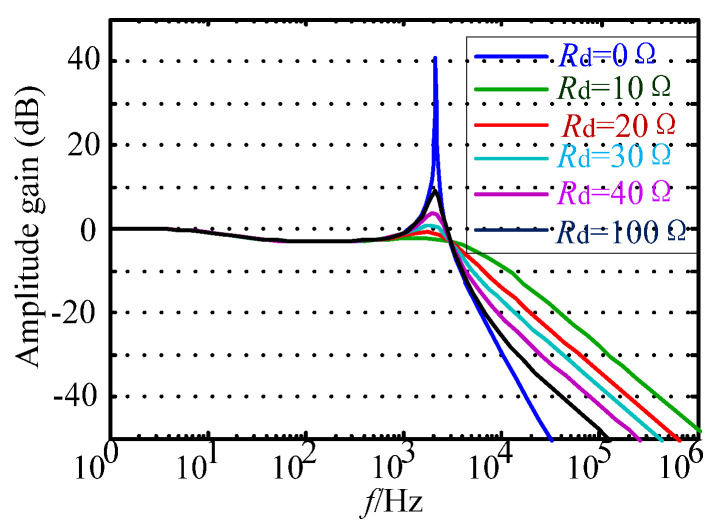
Bode diagram of conventional solution.

For the above damping control scheme, when the different damping resistors are selected, the system resonance phenomenon caused by the LC filter can be effectively realized. The Bode diagram of the traditional passive damping system is shown in Figure 4.

According to the Bode diagram, when the system is in an undamped state, the gain of the LC filter at the resonant frequency is as high as 40 dB. There is also a significant resonance peak, which will seriously affect the normal operation of the system. As the damping resistance increases, the system resonance peak decreases significantly. When *R*_d_ is equal to 10 Ω, the filter gain is reduced to −2.5 dB; when *R*_d_ is equal to 30 Ω, the filter gain is reduced by about 0 dB; when *R*_d_ is equal to 100 Ω, the filter gain is increased to 9 dB. Therefore, it can be concluded that as the damping resistor *R*_d_ increases continuously, the gain of the filter at the resonant frequency rises remarkably, resulting in a significant oscillation of the system. In addition, at high frequencies around the switching frequency, the smaller the damping resistor *R*_d_, the weaker the high-frequency harmonic attenuation capability, resulting in an increase in the harmonic content of the alternating current, which seriously affects the quality of the alternating current. Moreover, the traditional passive damping scheme also has the disadvantage that the system damping power loss is large.

**Figure 5:**
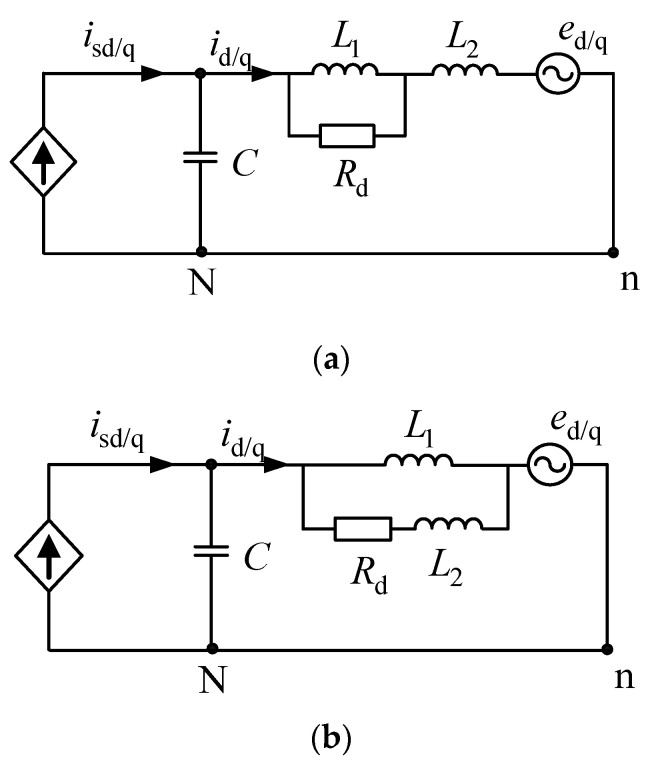
Proposed split-inductor passive damping topology. (**a**) Solution 1; (**b**) Solution 2.

In order to solve the shortcomings of the damping scheme of the traditional inductive shunt resistor, this paper proposes a new passive damping scheme for split inductors, as shown in Figure 5.

According to Figure 5, the new passive damping filter transfer function of solution 1 and solution 2 can be separately calculated.

(10) $${\frac{i_{d/q}(s)}{i_{\mathrm{sd}/q}(s)}}_{1}=\frac{L_{1}s+R_{d}}{L_{2}L_{1}Cs^{3}+\left(L_{2}+L_{1}\right)CR_{d}s^{2}+L_{1}s+R_{d}}$$

(11) $${\frac{i_{d/q}(s)}{i_{\mathrm{sd}/q}(s)}}_{2}=\frac{\left(L_{2}+L_{1}\right)s+R_{d}}{L_{2}L_{1}Cs^{3}+L_{1}CR_{d}s^{2}+\left(L_{2}+L_{1}\right)s+R_{d}}$$

### 3.2. Analysis of Proposed Damping Scheme Based on Split Inductors

**Figure 6:**
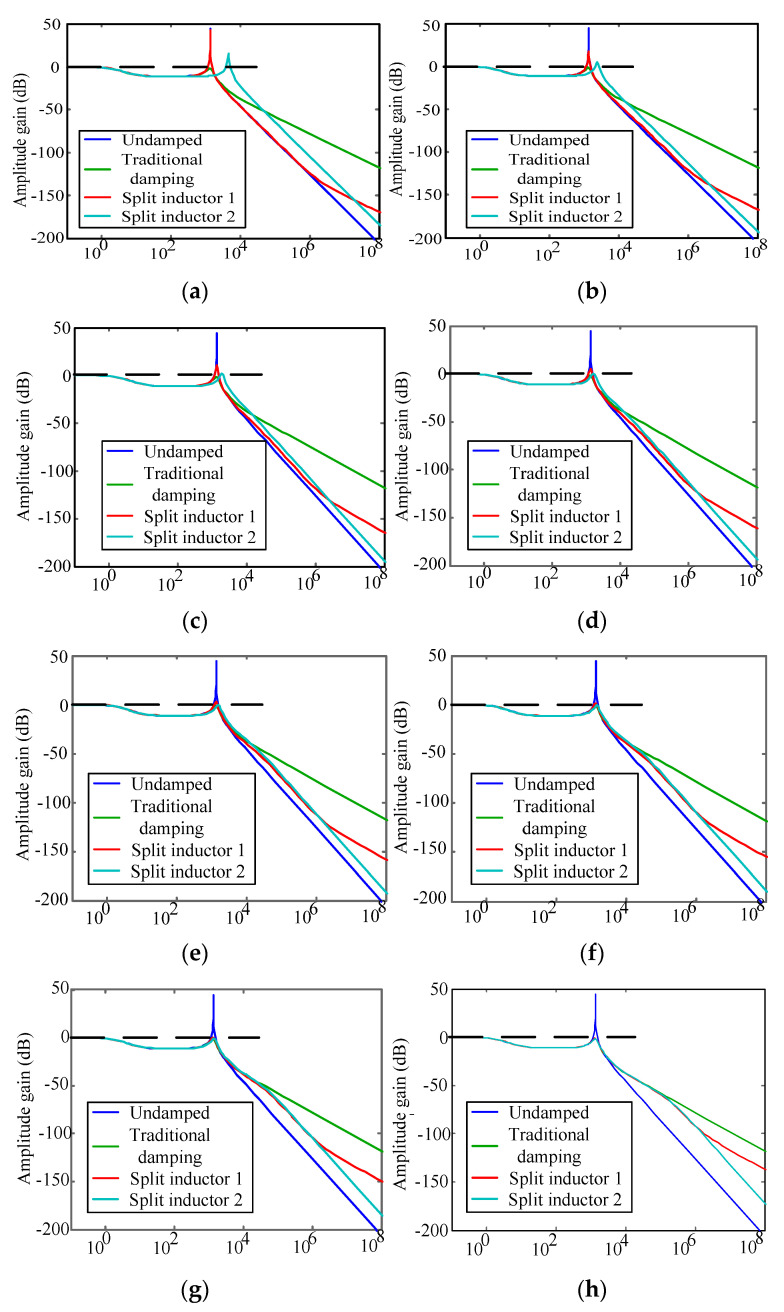
Comparison of system gains at different split inductor ratios. (**a**) *n* = 0.1, f/Hz; (**b**) *n* = 0.5, f/Hz; (**c**) *n* = 1, f/Hz; (**d**) *n* = 2, f/Hz; (**e**) *n* = 3, f/Hz; (**f**) *n* = 5, f/Hz; (**g**) *n* = 10, f/Hz; (**h**) *n* = 50, f/Hz.

Under the condition that the filter parameters of the traditional damping scheme are kept unchanged, the inductors are split and two new passive damping topologies can be obtained, respectively, which is shown in Figure 5. The parameters of the traditional passive damper filter are as follows: the filter capacitance is 9.4 µF, the filter inductor is 2 mH, and the damper resistance is equal to 50 Ω. When the sum of the filter inductances of the new split inductor scheme remains unchanged (*L* = *L*_1_ + *L*_2_), the split inductor ratio is *n = L*_1_/*L*_2_. According to (9)–(11), when different ratios *n* are taken, the Bode diagrams of the three passive damping filter transfer functions are plotted, which is shown in Figure 6.

According to the gain comparison analysis, when the split inductor ratio *n* is less than 1, the gain of the two split inductor damping schemes at the resonant frequency is significantly greater than 0 dB. Compared with the traditional passive damping scheme, this kind of situation can not achieve the purpose of effectively suppressing the resonance phenomenon of the system. However, according to Figure 6, when the split inductor ratio *n* is greater than 1, the attenuation capability of the high frequency harmonics of the split inductor damping scheme is almost the same as that of the undamped scheme, which is far superior to the conventional damping scheme. When the split inductor ratio *n* is greater than or equal to 1, the system resonance gain spike is less than 0 dB as *n* increases, and almost the same resonance suppression effect can be achieved compared to the conventional passive damping scheme. Moreover, when the frequency is less than or equal to 100 kHz, the attenuation capability of the system’s high-frequency harmonics is almost the same as that of an undamped system.

### 3.3. Power Loss Analysis of Proposed Scheme

For the system power loss of the traditional passive damping control scheme and the proposed novel passive damping control scheme of the split inductor, the power losses of the three main system frequencies are analyzed, including the fundamental frequency, the system resonant frequency and the switching frequency.

According to Figure 3, when the system power, DC measurement input and AC-side grid voltage are kept constant, and without considering the line resistance, the active power consumed by the system damping resistor of the conventional solution and the reactive power absorbed by the filter inductor L can be obtained, which is shown in (12). Where the magnitude of the series inductor-side voltage effective value is *u* = *u*_c_ − *u*_g_.

(12) $$\left\{ \begin{array}{l} P_{{0\text{\_}R}_{d}}=\frac{u^{2}}{R_{d}}\\ Q_{0\text{\_}L}=\frac{u^{2}}{\omega *L} \end{array}\right.$$

Since the sum of the filter inductors of the two new split inductor schemes remains the same (*L* = *L*_1_ + *L*_2_), the split inductor ratio is *n = L*_1_/*L*_2_. According to Figure 5, the active power consumed by the damping resistors of the two split inductor schemes is shown in (13).

(13) $$\left\{ \begin{array}{l} P_{{1\text{\_}R}_{d}}=\frac{u^{2}}{R_{d}}\left(\frac{L_{1}^{2}R_{d}^{2}}{L_{1}^{2}L_{2}^{2}\omega^{2}+L_{1}^{2}R_{d}^{2}+2L_{1}L_{2}R_{d}^{2}+L_{2}^{2}R_{d}^{2}}\right)\\ P_{{2\text{\_}R}_{d}}=\frac{u^{2}}{R_{d}}\left(\frac{R_{d}^{2}}{L_{2}^{2}\omega^{2}+R_{d}^{2}}\right) \end{array}\right.$$

The reactive power consumed by the inductors of the two split inductor schemes is shown in (14).

(14) $$\left\{ \begin{array}{l} Q_{1\text{\_}L1\text{\_}L2}=\frac{u^{2}}{\omega }*\frac{L_{1}R_{d}^{2}+L_{2}(L_{1}^{2}\omega^{2}+R_{d}^{2})}{L_{1}^{2}L_{2}^{2}\omega^{2}+L_{1}^{2}R_{d}^{2}+2L_{1}L_{2}R_{d}^{2}+L_{2}^{2}R_{d}^{2}}\\ Q_{2\text{\_}L1\text{\_}L2}=\frac{u^{2}}{\omega L_{1}}+\frac{u^{2}*\omega L_{2}}{L_{2}^{2}\omega^{2}+R_{d}^{2}} \end{array}\right.$$

According to (12)–(14), the ratios of the two new split inductance damping schemes and the conventional solutions on the sum of the active power and the inductor-consuming reactive power of the damping resistor consumption system can be obtained, respectively, which is shown in (15)–(17).

(15) $$\lambda_{1}=\frac{P_{{1\text{\_}R}_{d}}+Q_{1\text{\_}L1\text{\_}L2}}{P_{{0\text{\_}R}_{d}}+Q_{0\text{\_}L}}=\frac{\omega LR_{d}}{\omega L+R_{d}}*\frac{L_{1}^{2}R_{d}+\frac{R_{d}^{2}L_{1}+L_{2}L_{1}^{2}\omega^{2}+L_{2}R_{d}^{2}}{\omega }}{L_{1}^{2}L_{2}^{2}\omega^{2}+L_{1}^{2}R_{d}^{2}+2L_{1}L_{2}R_{d}^{2}+L_{2}^{2}R_{d}^{2}}$$

(16) $$\lambda_{2}=\frac{P_{{2\text{\_}R}_{d}}+Q_{2\text{\_}L1\text{\_}L2}}{P_{{0\text{\_}R}_{d}}+Q_{0\text{\_}L}}=\frac{\omega LR_{d}}{\omega L+R_{d}}*\left(\frac{R_{d}+\omega L_{2}}{L_{2}^{2}\omega^{2}+R_{d}^{2}}+\frac{1}{\omega L_{1}}\right)$$

(17) $$\lambda_{3}=\frac{\lambda_{2}}{\lambda_{1}}=\frac{L_{2}^{2}\omega^{2}+R_{d}^{2}+L_{1}\omega \left(L_{2}\omega +R_{d}\right)}{L_{1}\left(L_{2}^{2}\omega^{2}+R_{d}^{2}\right)}*\frac{L_{1}^{2}L_{2}^{2}\omega^{2}+L_{1}^{2}R_{d}^{2}+2L_{1}L_{2}R_{d}^{2}+L_{2}^{2}R_{d}^{2}}{L_{1}^{2}L_{2}\omega^{2}+L_{1}^{2}R_{d}\omega +\left(L_{1}+L_{2}\right)R_{d}^{2}}$$

**Figure 7:**
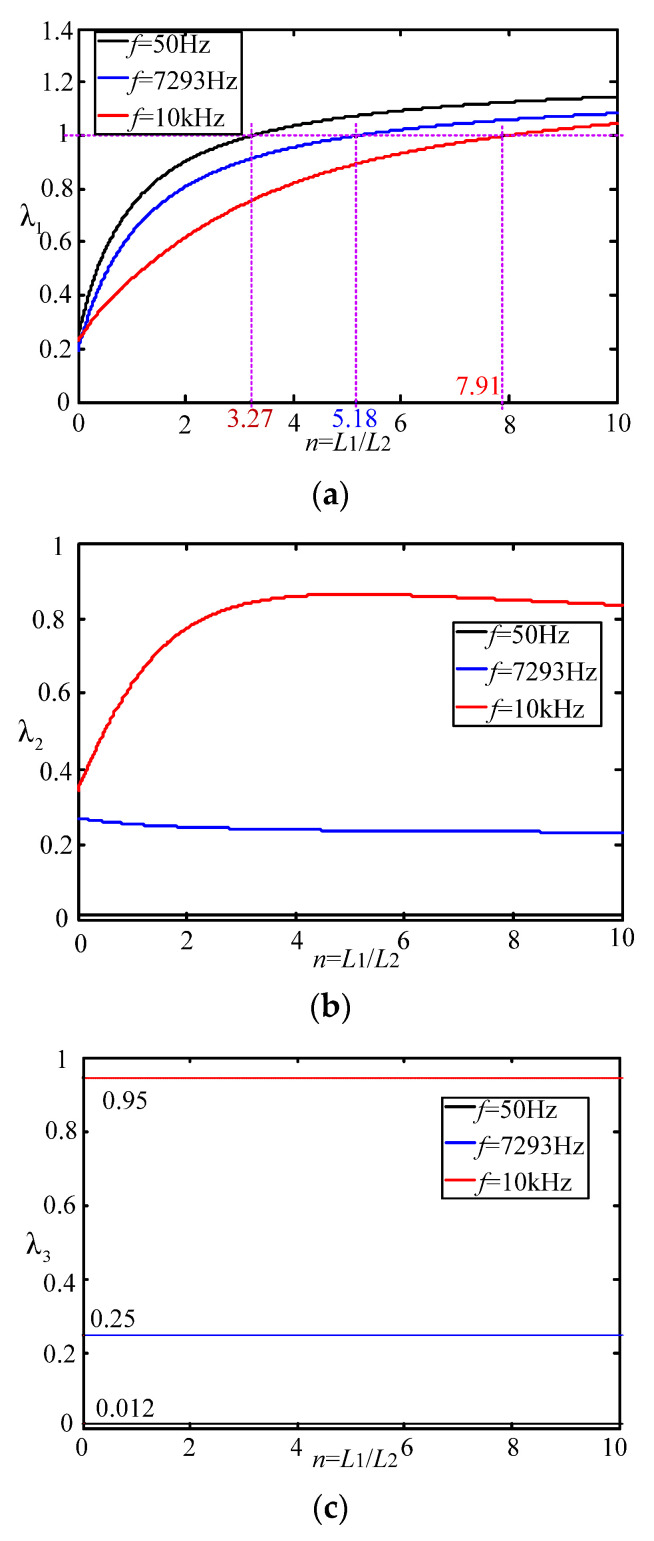
Power dissipation ratio analysis for different split inductor ratios. (**a**) Power consumption ratio of split inductor solution 1 and conventional solution; (**b**) Power consumption ratio of split inductor solution 2 and conventional solution; (**c**) Power consumption ratio of split inductor solution 2 and solution 1.

According to the theoretical design, the three key frequencies under the LC filter system are the fundamental frequency of 50 Hz, the resonant frequency of 7293 Hz and the switching frequency of 10 kHz. In the case of considering only these three frequencies, the MATLAB software can be used to plot the power dissipation ratio λ of the split inductor value *n* under different values, which is shown in Figure 7.

According to Figure 7a, when the split inductor ratio *n* > 3.27 and $\lambda_{1}$ > 1, the power consumption of the proposed solution 1 is higher than that of the conventional solution at the fundamental frequency (see black line). 

When the split inductor ratio *n* > 5.18 and $\lambda_{1}$ > 1, the power consumption of the proposed solution 1 is higher than that of the conventional solution at the system resonant frequency (see blue line).

When the split inductor ratio *n* > 7.91 and $\lambda_{1}$ > 1, the power consumption of the proposed solution 1 is higher than that of the conventional solution at the switching frequency (see red line).

In summary, at three critical frequencies, in order to ensure that the system damping power of the split inductor solution 1 is smaller than that of the conventional solution, the split inductor ratio is equal to 3.27.

According to Figure 7b, at three key frequencies, the split inductor ratio can be arbitrarily determined to ensure that the system damping power of the split inductor solution 2 is smaller than the system damping power of the conventional damping scheme. According to Figure 7c, at three critical frequencies, regardless of the split inductor ratio, the system damping power of the split inductor damping solution 2 will always be smaller than the system damping power of the split inductor damping solution 1.

### 3.4. Stability Analysis of Proposed Scheme with Different Damping Resistances

**Figure 8:**
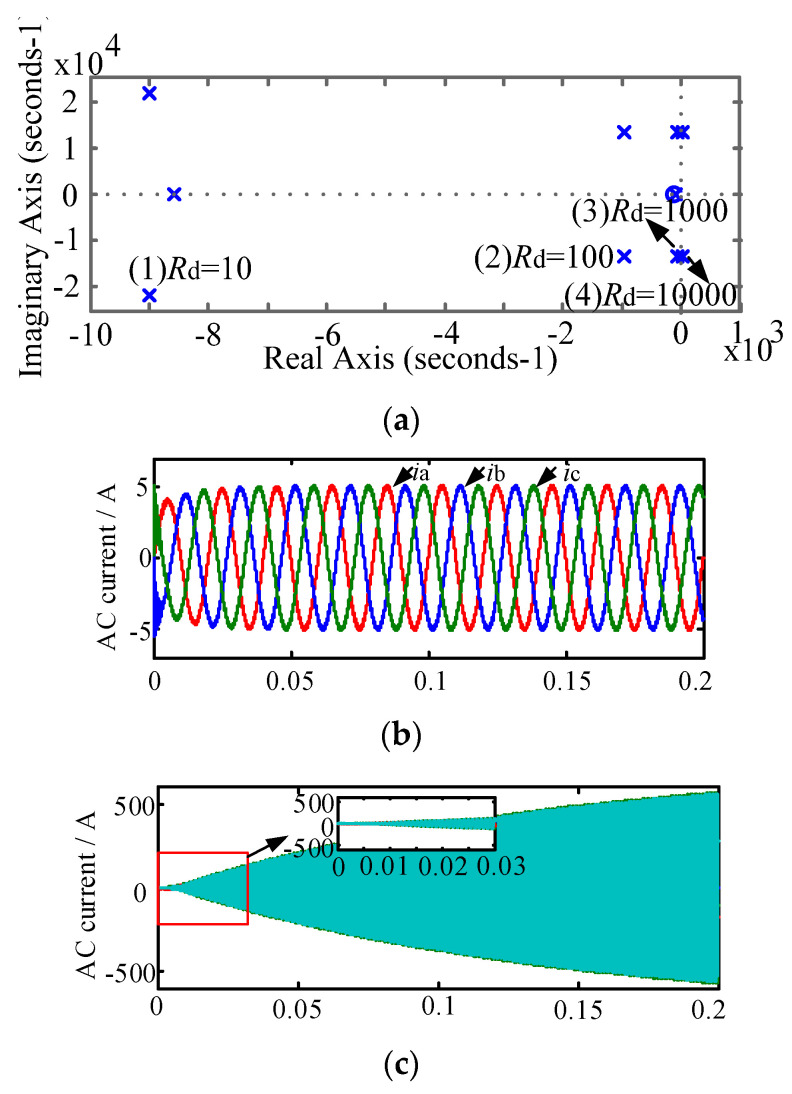
System stability analysis of split-inductive damping solution 1. (**a**) AC current with damping resistance of 100; (**b**) AC current with damping resistance of 100; (**c**) AC current with damping resistance of 10,000.

**Figure 9:**
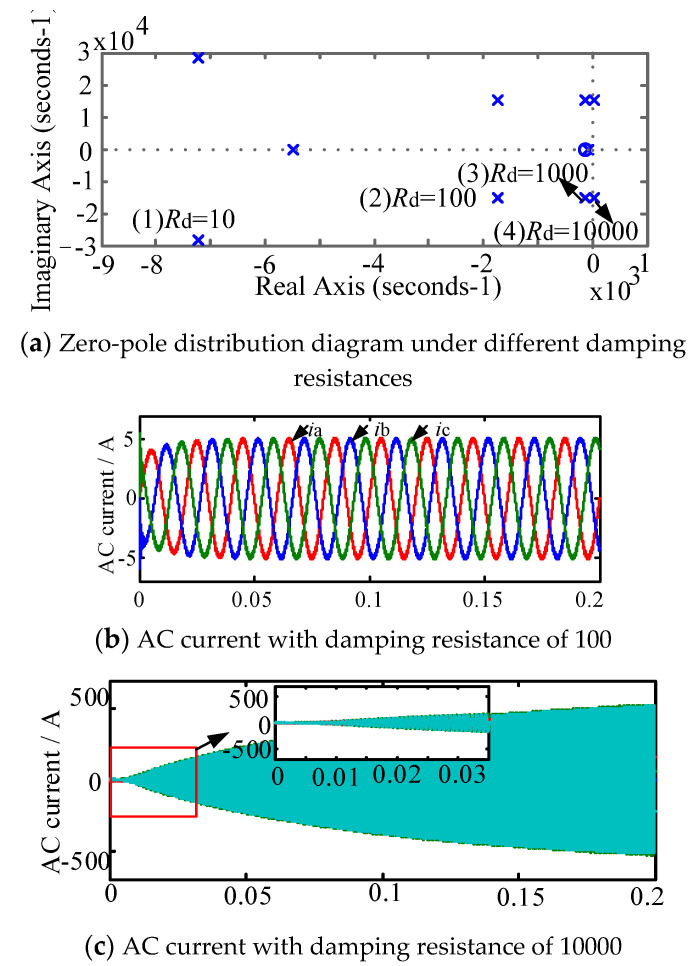
System stability analysis of split-inductive damping solution 2. (**a**) Zero-pole distribution diagram under different damping resistances; (**b**) AC current with damping resistance of 100; (**c**) AC current with damping resistance of 10,000.

According to the theoretical analysis of the previous section, when the new splitting inductance value is 3, the damping effect and the power loss are small. This section mainly studies the stability of the system when the damping resistance is equal to different values, which is shown in Figure 8 and Figure 9.

According to Figure 7 and Figure 8, when the split inductor ratio is 3 and the damping resistance is 10 Ω, 100 Ω and 1000 Ω, respectively, the system transfer function poles are all in the left half plane of the S plane, indicating that both new schemes can keep the system stable. However, when the damping resistance is 10,000 Ω, the system pole is located in the right half plane of the S plane, indicating that the system is in an unstable state. Figure 8b,c and Figure 9b,c are system simulation comparisons of the two split-inductance damping schemes with damping resistance values of 100 Ω and 10,000 Ω, respectively. From the comparison chart, it can be concluded that the system pole distribution and theoretical analysis are consistent.

In summary, it can be concluded that when the split-inductance ratio is in the range of 1 ≤ *n* ≤ 3.27, the novel scheme proposed in this paper can not only ensure the damping effect of the system but also reduce the power loss of the system.

## 4. Simulation and Experimental Results

In order to verify the correctness of the proposed new passive damping scheme, the simulation and experimental verification of the new scheme are carried out. The proposed new passive damping scheme is simulated and analyzed. The simulation parameters are shown in Table 1.

**Figure 10:**
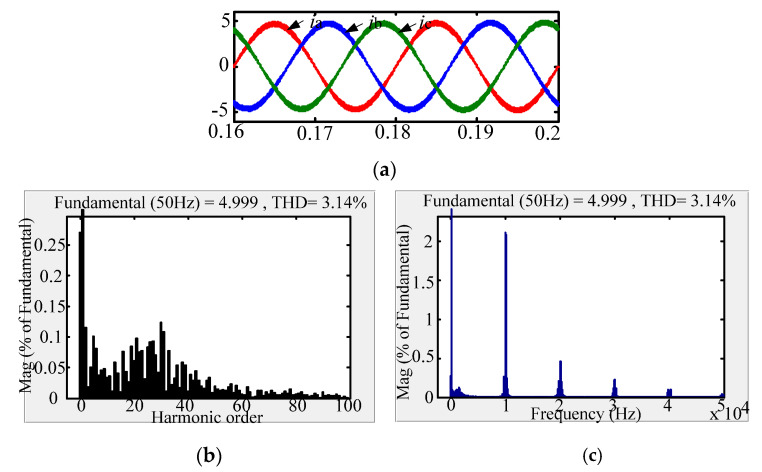
AC-side current waveform of traditional passive damping scheme. (**a**) AC-side current; (**b**) Low-frequency harmonic FFT analysis; (**c**) High-frequency harmonic FFT analysis.

**Figure 11:**
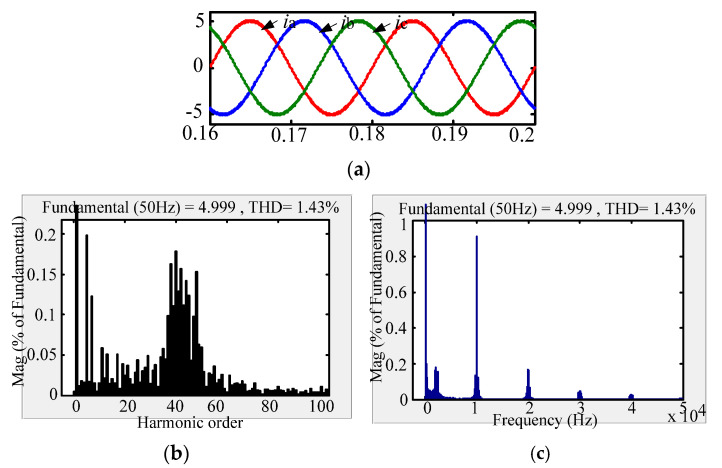
AC-side current waveform of split inductor damping solution 1. (**a**) AC-side current; (**b**) Low-frequency harmonic analysis; (**c**) High-frequency harmonic analysis.

**Figure 12:**
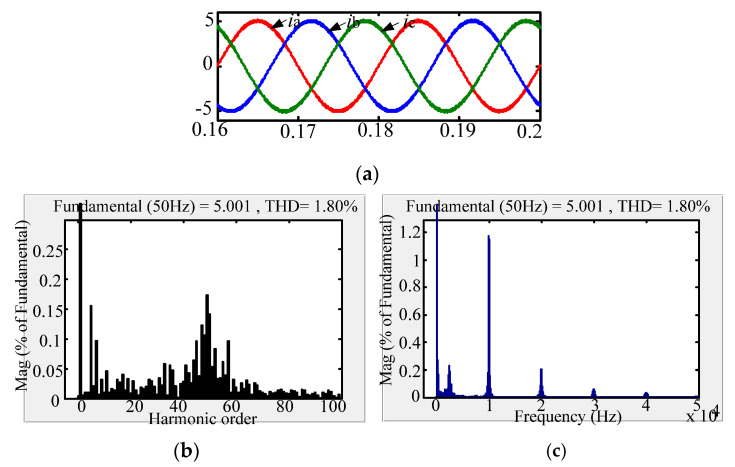
AC-side current waveform of split inductor damping solution 2. (**a**) AC-side current; (**b**) Low-frequency harmonic FFT analysis; (**c**) High-frequency harmonic FFT analysis.

The comparison between the new passive damping scheme and the traditional passive damping scheme with respect to the AC-side current FFT simulation waveform is shown in Figure 10, Figure 11 and Figure 12. According to the simulation comparison chart, there are many higher harmonics in the AC-side current waveform of the conventional solution. According to the FFT analysis, the THD of the grid-side current of the conventional solution is 3.14%. Compared with the traditional passive damping scheme, the AC-side currents of the two new passive damping schemes only have lower harmonics. According to the FFT analysis, the THD of the AC-side current of the split inductor damping solution 1 is 1.43%; the THD of the AC-side current of the split inductor damping solution 2 is 1.80%. According to the above analysis, the current quality of the AC side of the proposed scheme is more advantageous.

Comparing the AC-side current low-frequency harmonic FFT analysis of the three schemes, it can be concluded that the low-order harmonic content of the three schemes meets the current harmonic content of the international grid. In addition, the three damping schemes have almost the same system gain at low-order frequencies and resonant frequencies, which is consistent with theoretical analysis. Comparing the AC-side current harmonic content of the two new damping schemes at the low-order frequency, the AC-side current quality of the split-inductive damping solution 2 is better.

Comparing the AC-side current high frequency harmonic FFT analysis of the three damping schemes, at the switching frequency and the integer multiple frequency, the harmonic content of the proposed scheme is more than double that of the conventional solution. Moreover, it is equal to the gain of the high-order frequency of the undamped system, which is consistent with the theoretical analysis. Comparing the two-side damping scheme with the grid-side current harmonic content at the system switching frequency and the integer multiple frequency, the split-side inductance damping solution 1 has better grid-side current quality.

In summary, in the case of selecting the appropriate split-inductance ratio, the proposed damping scheme of the split inductor can not only achieve the same damping effect as the traditional damping scheme, but also reduce the system damping power consumption. Comparing the two new damping schemes, selecting the appropriate split-inductance ratio can reduce the system damping power consumption. Moreover, in the FFT analysis of AC current, the two schemes have complementary advantages in the harmonic content of low-order frequencies and high-order frequencies.

In order to further verify the effectiveness of the proposed scheme, the experimental platform was built in the laboratory. The experimental parameters are shown in Table 2. 

**Figure 13:**
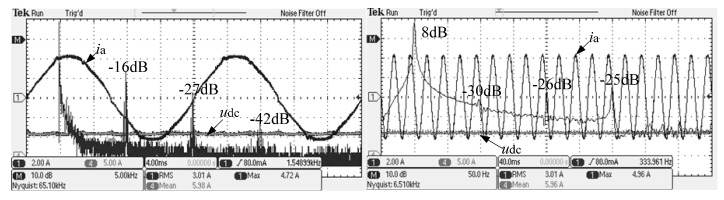
System AC current FFT analysis of traditional passive damping scheme.

**Figure 14:**
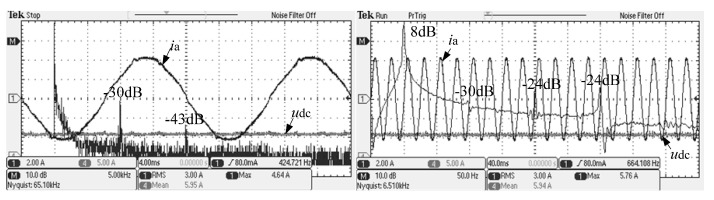
System AC current FFT analysis of the new split inductor solution 1.

**Figure 15:**
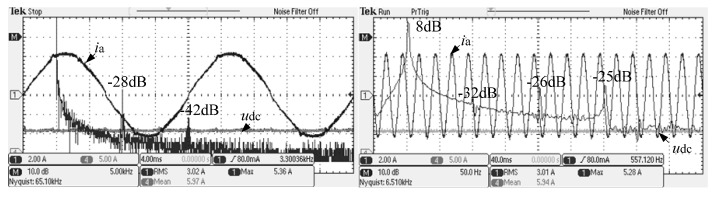
System AC current FFT analysis of the new split inductor solution 2.

Split inductor and the conventional passive damping scheme were compared with the FFT analysis of the alternating current. The experimental waveform is shown in Figure 13, Figure 14 and Figure 15.

From Figure 13, it can be observed that the system AC current gains of the conventional damping scheme are −16 dB, −27 dB and −42 dB, respectively, at integer frequencies such as the switching frequency and the double switching frequency. 

From Figure 14, it can be observed that, at the switching frequency and its integer frequency, the system AC current gain of the split inductor solution 1 is −30 dB and −43 dB, respectively.

From Figure 15, it can be observed that, at the switching frequency and its integer frequency, the system AC current gain of the split inductor solution 2 is −28 dB and −42 dB, respectively. It can be seen that in the case of the optimal split-inductance ratio, the new split-inductor passive damping scheme has higher harmonic attenuation capability, which is consistent with theoretical analysis. 

On the other hand, from Figure 13, Figure 14 and Figure 15, the low-order harmonic content of 3, 5 and 7 times the system alternating current of the new scheme and the conventional solution is almost the same, which is consistent with the theoretical analysis. 

In summary, the new split-inductor passive damping scheme has better AC current quality, which has advantages.

## 5. Conclusions

This paper has presented a novel damping control for the grid-connected converter based on the optimal split-inductor concept. The theoretical analysis and experimental results reveal that the proposed method can not only achieve almost the same damping effect as the conventional solution, but also reduce the power loss of the damping resistor. The experimental tests have been provided to verify the effectiveness of the proposed method.

## Figures and Tables

**Table 1:** Simulation parameter.

Parameter	Value	Parameter	Value
Direct current *I*_dc_	8 A	Carrier frequency *f*_c_	10 kHz
Grid voltage *E*	94 V	Fundamental frequency *f*_m_	50 Hz
Filter inductor *L*	2 mH	Filter capacitor *C*	9.4 μF
Damping resistor *R*_d_	50 Ω	P parameter	*k*_p_ = 0.046
Split inductor ratio *n*	3	I parameter	*k*_i_ = 2.383

**Table 2:** Experimental parameters.

Parameter	Value	Parameter	Value
Direct current *I*_dc_	6 A	Carrier frequency *f*_c_	10 kHz
Modulation frequency *f*_m_	50 Hz	Filter inductor *L*	2 mH
Filter capacitor *C*	9.4 μF	Damping resistor *R*_d_	51 Ω
P parameter	*k*_p_ = 0.046	Split inductor ratio *n*	3
I parameter	*k*_i_ = 2.383	Grid voltage *E*	47 V

## Data Availability

Not applicable.
